# Injuries to the Eye in Their Medico-Legal Aspect

**Published:** 1900-06

**Authors:** 


### *BOOK REVIEWS, PAMPHLETS, EXCHANGES.*

### *BOOK REVIEWS.*

[Contributions solicited for review.]

INJURIES TO THE EYE IN THEIR MEDICO-LEGAL ASPECT. By S. Baudry, M.D., Professor in the Faculty of Medicine, University of Lille, France, etc. Translated from the original by Alfred James Ostheimer, Jr., M.D., of Philadelphia, Pa. Revised and edited by Charles A. Oliver, A.M., M.D., Attending Surgeon to the Wills Eye Hospital; Ophthalmic Surgeon to the Philadelphia Hospital; Member of the American and French Ophthalmological Societies, etc. With an Adaptation of the Medico-Legal Chapter to the Courts of the United States of America, by Charles Sinkler, Esq., Member of the Philadelphia Bar. $5\frac{5}{8}$ x $7\frac{7}{8}$ inches. Pages x–161. Extra cloth, $1.00, net. The F. A. Davis Co., Publishers, 1914–16 Cherry St., Philadelphia, Pa.

This small brochure is an excellent little hand-book to be placed in the library of every ophthalmologist, and, we might say, likewise the general surgeon. It is a subject which has hitherto only been considered in isolated articles published in medical literature, and the compilation of the present volume will be a welcome visitor. The whole subject has been thoroughly discussed, and the arrangement of the chapters is most excellent. The American editor, Dr. Oliver, is to be congratulated. B.

